# Postoperative adjuvant chemotherapy and chemoimmunotherapy after radical resection for biliary tract cancer: a retrospective study

**DOI:** 10.1093/oncolo/oyaf163

**Published:** 2025-06-21

**Authors:** Yuhuai Peng, Guoyi Xia, Yufeng Li, Jia Zhou, Sulai Liu, Chuang Peng, Yuewei Tao, Ou Li, Yinghui Song

**Affiliations:** Central Laboratory, Hunan Provincial People’s Hospital/The First Affiliated Hospital of Hunan Normal University, Changsha, P. R. China; Department of Hepatobiliary Surgery, Hunan Provincial People’s Hospital/The First Affiliated Hospital of Hunan Normal University, Changsha, P. R. China; Department of Hepatobiliary Surgery, The Central Hospital of Shaoyang, Shaoyang, P. R. China; Department of Hepatobiliary Surgery, Hunan Provincial People’s Hospital/The First Affiliated Hospital of Hunan Normal University, Changsha, P. R. China; Department of Hepatobiliary Surgery, Hunan Provincial People’s Hospital/The First Affiliated Hospital of Hunan Normal University, Changsha, P. R. China; The First school of Clinical Medicine, Lanzhou University, Lanzhou, P. R. China; Central Laboratory, Hunan Provincial People’s Hospital/The First Affiliated Hospital of Hunan Normal University, Changsha, P. R. China; Department of Hepatobiliary Surgery, Hunan Provincial People’s Hospital/The First Affiliated Hospital of Hunan Normal University, Changsha, P. R. China; Department of Hepatobiliary Surgery, Hunan Provincial People’s Hospital/The First Affiliated Hospital of Hunan Normal University, Changsha, P. R. China; School of Medicine, University of Dundee, Ninewells Hospital, Dundee, UK; Department of Hepatobiliary Surgery, Hunan Provincial People’s Hospital/The First Affiliated Hospital of Hunan Normal University, Changsha, P. R. China; Central Laboratory, Hunan Provincial People’s Hospital/The First Affiliated Hospital of Hunan Normal University, Changsha, P. R. China; Department of Hepatobiliary Surgery, Hunan Provincial People’s Hospital/The First Affiliated Hospital of Hunan Normal University, Changsha, P. R. China

**Keywords:** adjuvant chemoimmunotherapy, adjuvant chemotherapy, biliary tract cancers, overall survival, recurrence-free survival

## Abstract

**Background and Objectives:**

The prognosis of biliary tract cancers (BTC) after radical resection is still unsatisfactory. However, the clinical value of adjuvant therapy remains controversial. This retrospective study aimed to evaluate the clinical value of adjuvant chemotherapy and adjuvant chemoimmunotherapy in patients with BTC after radical resection.

**Methods:**

Data from BTC patients who underwent radical resection were retrospectively obtained from Hunan Provincial People’s Hospital between January 2020 and July 2024. Patients were divided into observation group, adjuvant chemotherapy group, and adjuvant chemoimmunotherapy group according to the treatment received by the patient after surgery. Survival curves were determined by the Kaplan–Meier method. The COX proportional hazards regression model was used to determine independent prognostic risk factors. The adjuvant chemotherapy group and adjuvant chemoimmunotherapy group were analyzed by PSM at a 1:1 ratio.

**Results:**

A total of 219 patients with BTC were reenrolled in this study, with 108 cases of iCCA, 39 cases of pCCA, 15 cases of DCCA, and 57 cases of GBC. Eighty-seven patients (39.73%) received surgery alone, 69 patients (31.51%) received postoperative adjuvant chemotherapy, and 63 patients (28.77%) received postoperative adjuvant chemoimmunotherapy. There was no different significance for median recurrence-free survival (RFS) in the 3 groups (13.20 vs 20.40 vs 19.68 months; *P* = .195). The median overall survival (OS) was the longest in the chemoimmunotherapy group (29.20 vs 31.5 vs 43.27 months; *P* = .003). After propensity score matching (PSM), there was no difference in median RFS in the 2 adjuvant groups (22.03 vs 19.87 months; *P* = .350). The median OS was longer in the chemoimmunotherapy group (45.27 vs 29.40 months; *P* = .015). In Cox analysis, lymph node metastasis, differentiation, and adjuvant treatment were the independent predictors of OS in patients with BTC. The most common adverse events were of any grade of hematologic toxicity. No drug-related deaths occurred in either group.

**Conclusions:**

The safety of chemoimmunotherapy was acceptable and could significantly prolong the overall survival of BTC. These data provided a basis for an additional prospective clinical trial to evaluate the efficacy of chemoimmunotherapy in adjuvant therapy for BTC.

**Implications for Practice**

This retrospective study evaluated the clinical value of adjuvant chemotherapy and adjuvant chemoimmunotherapy in patients with BTC after radical resection. The safety of chemoimmunotherapy was acceptable and could significantly prolong the overall survival of BTC.

## Introduction

Biliary tract cancers (BTC) are highly heterogeneous diseases that can be classified as cholangiocarcinoma (intrahepatic cholangiocarcinoma, hilar cholangiocarcinoma, extrahepatic cholangiocarcinoma) and gallbladder carcinoma according to the anatomical origin of the tumor site.^1^ The incidence of BTC worldwide has significant regional differences and is increasing year by year.^2^ BTC is characteristic of insidious onset, and most patients are already in the advanced stage when diagnosed. The proportion of patients who can undergo curative surgery is relatively low.^3^ Even with radical resection surgery, the rate of recurrence of BTC is still relatively high.^4^ This is closely related to the biological characteristics of BTCs, as the onset is insidious and the stage is late when seeking medical attention, and it is prone to lymph node metastases, microvascular invasion, nerve invasion, etc.^5^ Multiple studies have shown that postoperative adjuvant therapy can improve prognosis. The BILCAP study and the ASCOT study showed that patients undergoing radical resection of BTCs can benefit from adjuvant therapy with capecitabine and S-1, respectively.^6,7^ A multicenter study on the value of postoperative adjuvant therapy for cholangiocarcinoma also suggests that postoperative adjuvant therapy is particularly necessary for patients with risk factors.^8^ For early tumors such as T1N0M0, postoperative adjuvant therapy can also prolong the prognosis of patients compared to the observation group.^9^ There have been attempts to combine adjuvant chemotherapy with gemcitabine and cisplatin compared to capecitabine in the treatment of lymph node-positive extrahepatic bile duct cancer. Combination therapy was found to not improve survival outcomes.^10^ There are still too many issues to address regarding postoperative adjuvant therapy for BTC and clinical evidence on the use of targeted drugs or immune checkpoint inhibitors for postoperative adjuvant therapy for BTC is even more limited.

Immunotherapy, together with targeted therapy, has emerged as a new treatment modality and is widely used in HCC. It is mainly in the clinical trial stage in BTC.^11^ Currently, targeted and immunotherapy methods are mainly focused on advanced patients with BTC. For advanced patients with BTC, targeted combined immunotherapy and chemotherapy have achieved a survival improvement.^12,13^ This also provides some basis for the application of this treatment regimen in adjuvant therapy in patients with BTC. A study comparing the efficacy and safety of adjuvant therapy between chemotherapy and chemotherapy combined with immunotherapy in 101 postoperative patients with cholangiocarcinoma showed that the combination of chemotherapy with immunotherapy extends the recurrence-free survival (RFS) and OS of patients with cholangiocarcinoma after radical resection compared to chemotherapy alone.^14^

Based on the issues and challenges of postoperative adjuvant therapy of BTC, this study retrospectively analyzed the postoperative treatment of patients with BTC admitted to Hunan Provincial People’s Hospital. By comparing the prognostic effects of postoperative treatments, including observation, chemotherapy, and adjuvant chemoimmunotherapy in patients with BTC after radical surgery, our objective is to provide new clinical evidence for the selection of postoperative adjuvant therapy for BTC.

## Materials and methods

### Study design and patients

Between January 2020 and December 2024, consecutive patients with histologically confirmed BTC who received radical resection were screened at Hunan Provincial People’s Hospital. The observation group is defined as those who have not received any antitumor treatment after surgery and who only undergo regular follow-up examinations. The chemotherapy group is defined as the regular completion of at least 6 cycles of adjuvant chemotherapy after surgery. The adjuvant chemoimmunotherapy group is defined as receiving a combination of immunotherapy and chemotherapy for at least 6 cycles after surgery. The exclusion criteria for this cohort study were the following: (1) incomplete clinical data, (2) mortality within 1 month after surgery, (3) patients with other malignant tumors or autoimmune diseases; patients with a history of chemotherapy or immunotherapy. This study was carried out according to the Declaration of Helsinki and was approved by the Ethics Committee of Hunan Provincial People’s Hospital (NO. 2023-156).

### Treatment and data collection

The chemotherapy regimen consisted of gemcitabine plus cisplatin (21.21%), capecitabine (14.39%), S-1 (47.73%), and fluorouracil plus oxaliplatin (16.67%). PD-1/L1 inhibitors were administered every 3 weeks. The dosage was determined based on the application of various drugs, including tislelizumab (30.16%), camrelizumab (20.63%), sintilimab (19.05%), pembrolizumab (17.46%), and toripalimab (12.70%). The dosing regimen for these drugs was tailored by clinical oncologists according to individual circumstances, taking into account tumor dynamics and patient-specific drug responses.

All relevant information, including age, sex, CA19-9, CEA, tumor size, tumor type, stage of TNM, stage of T, lymph node metastasis, microvascular invasion, perineural invasion, margin status, and differentiation, was collected by a detailed review of medical records.

### Follow-up and definition of endpoints

All patients were periodically followed every 2-3 months in the first 2 years after resection and then once every 6 months. Recurrence or metastasis was defined as the appearance of new lesions with the same radiological characteristics of BTC, and additional treatment was started immediately when the recurrence was confirmed. The treatment methods for recurrence included palliative symptomatic treatment, secondary postoperative treatment, drug therapy, interventional therapy, etc. The primary endpoint of this study was overall survival (OS), and the secondary endpoint was RFS. OS was calculated from the date of resection to the date of death or the latest follow-up. The RFS was defined as from the time of resection to the time of recurrence or from the date of the last follow-up.

### Statistical analysis

The analyses were performed using IBM SPSS 25.0 software (version 27.0). Continuous variables were all re-defined as categorical variables; hence, they were all evaluated using a Chi-square test or Fisher’s exact test. Survival curves were determined using the Kaplan–Meier method. The proportional hazards regression model of COX was used to determine independent prognostic risk factors. Statistical significance was defined as a *P*-value <.05.

## Results

### Baseline characteristics

**Figure 1.**
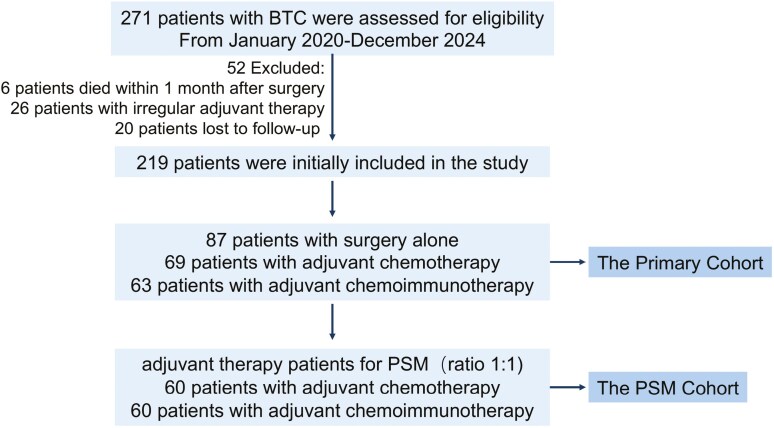
Flow chart of patients’ enrollment.

Two hundred seventy-one patients with BTC underwent resection. Fifty-two patients were excluded from the study for the following situation: 6 patients (2.21%) died within 1 month after surgery; 26 patients (9.59%) received irregular adjuvant therapy; and 20 patients (7.38%) lost follow-up. The patient enrollment flow chart is shown in Figure 1. The baseline characteristics of the 219 patients analyzed in the study are shown in Table 1. There were 137 patients (62.56%) over 60 years old and 124 patients (56.62%) were female. In this study, 108 cases of iCCA, 39 cases of pCCA, 15 cases of DCCA, and 57 cases of muscle-invasive or exceeding muscle-invasive GBC were included. One hundred forty-three patients (65.30%) had tumors with a diameter greater than 3 cm and 203 patients (92.69%) had a single tumor. One hundred thirty-four patients (61.87%) had stage I + II. Sixty-one patients (27.85%) had lymph node metastasis. One hundred eighteen patients (53.88%) had poorly differentiated and undifferentiated tumors. Eighty-seven patients (39.73%) only underwent surgery and were regularly followed after surgery (observation group). Sixty-nine patients (31.51%) received postoperative adjuvant chemotherapy, which included treatment with S-1 or gemcitabine combined with cisplatin, and completed at least 6 cycles of treatment (adjuvant chemotherapy group). Sixty-three patients (28.77%) received postoperative adjuvant chemotherapy combined with immunotherapy and completed at least 6 cycles of treatment (adjuvant chemoimmunotherapy group). See Table 1 for details.

**Table 1.** The baseline patient characteristics of 219 BTC patients.

Variables	All patients (*N* = 219)	Observation (*N* = 87)	Adjuvant chemotherapy (*N* = 69)	Adjuvant chemoimmunotherapy (*N* = 63)	*P*-value
*Age, years*					.281
≤60	82	27	29	26	
>60	137	60	40	37	
*Sex*					.689
Male	95	35	33	27	
Female	124	52	36	36	
*Tumor location*					.335
iCCA	108	40	32	36	
pCCA	39	10	16	11	
dCCA	15	4	6	5	
GBC	57	33	15	11	
*CA19-9, U/mL*					.127
≤37	105	42	34	29	
>37	114	45	35	34	
*CEA, ng/mL*					.262
≤5	161	59	52	50	
>5	58	28	17	13	
*Tumor size, cm*					.319
≤3	76	33	19	24	
>3	143	54	50	39	
*Tumor number*					.269
Single	203	82	61	60	
Multiple	16	5	8	3	
*TNM stage*					.362
I + II	134	58	41	35	
III	85	29	28	28	
*T stage*					.196
1 + 2	165	71	48	46	
3 + 4	54	16	21	17	
*Lymph node metastasis*					.106
No	158	66	53	39	
Yes	61	21	16	24	
*Microvascular invasion*					.930
No	169	66	54	49	
Yes	50	21	15	14	
*Perineural invasion*					.490
No	117	50	37	30	
Yes	102	37	32	33	
*Differentiation*					.461
Well/moderate	101	44	28	29	
Poor/undifferentiated	118	43	41	34	
*Margin status*					.381
R0	179	69	60	50	
R1	40	18	9	13	

### Survival outcomes

The survival outcomes of all patients were estimated. The median time of clinical follow-up was 20.43 (95% CI: 13.69-23.17) months. The 1-year, 2-year, and 3-year RFS rates of the entire group were 61.7%, 42.6%, and 33.6%, respectively. The 1-, 2-, and 3-year OS rates of the entire group were 74.3%, 61.9%, and 54.3%, respectively (Supplementary Figure 1A, B).

Although the BILCAP and ASCOT studies have revealed the value of adjuvant therapy for postoperative patients with BTC.^6,7^ There was still a significant portion of patients (87/219) in the observation group who did not receive postoperative adjuvant therapy. Patients who did not receive formal adjuvant therapy after surgery were treated with folk Chinese medicine for economic reasons, poor tolerance to chemotherapy, consideration of their physical condition, etc.

**Figure 2.**
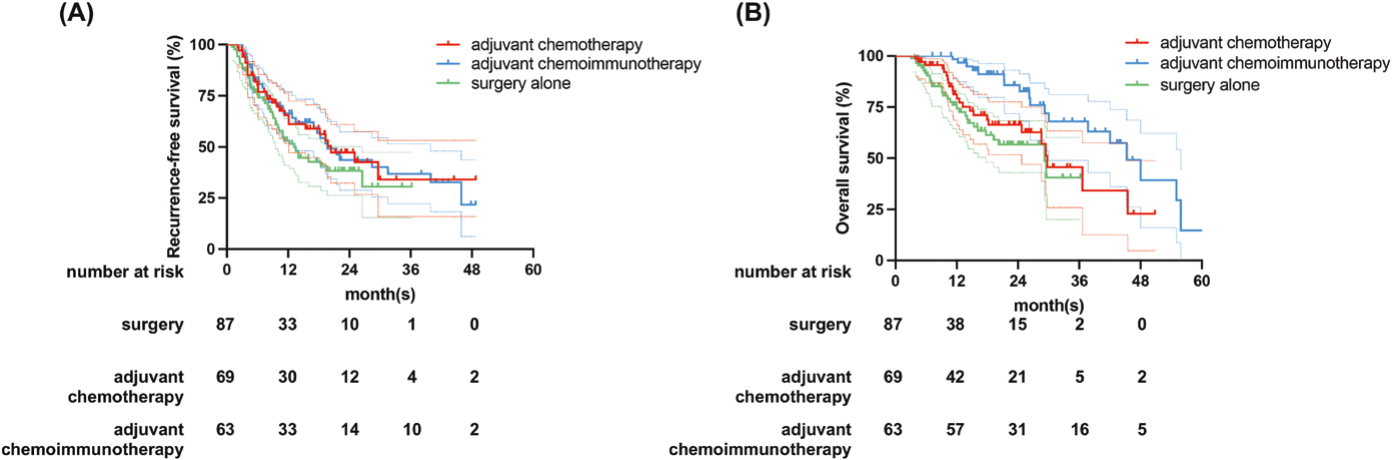
Kaplan–Meier curves for RFS (A) and OS (B) in the primary cohort of surgery alone, adjuvant chemotherapy and adjuvant chemoimmunotherapy.

The median RFS was 13.20 (95% CI: 10.09-16.31) months for the observation group, 20.40 (95% CI 13.10-27.71) for the adjuvant chemotherapy group, and 19.68 (95% CI: 13.86-23.01) months for the adjuvant chemoimmunotherapy group, respectively (*P* = .199). The median OS was 29.20 (95% CI: 18.67-39.73) months for the observation group, 31.5 (95% CI: 18.78-44.22) for the adjuvant chemotherapy group, and 43.27 (95% CI: 37.61-52.96) months for the adjuvant chemoimmunotherapy group, respectively (*P* = .003; Figure 2A, B). When comparing the adjuvant chemotherapy group to the observation group, there was significant improvement in RFS (*P* = .038) but not OS (*P* = .209). When comparing the adjuvant chemoimmunotherapy group to the observation group, there was significant improvement in RFS (*P* = .046) and OS (*P* = .007). Adjuvant chemotherapy appeared to have no significant effect on improving OS compared to the observation group. Furthermore, a propensity score matching (PSM) was performed between the observation group and the chemotherapy group and included 60 pairs of patients in a 1:1 ratio for survival comparison. It was found that the OS of the chemotherapy group was significantly longer than that of the group without adjuvant chemotherapy. The median OS was 27.60 (95% CI: 24.04-31.28) months for the observation group, and 35.05 (95% CI: 28.71-40.48) for the adjuvant chemotherapy group (*P* = .034; Supplementary Figure 2).

Next, a combination of adjuvant chemotherapy and adjuvant chemoimmunotherapy group in the adjuvant group compared to the observation group. The median RFS was 13.20 (95% CI: 10.09-16.31) months for the observation group, and 19.96 (95% CI: 14.08-25.42) for the adjuvant therapy group (*P* = .112). The median OS was 29.20 (95% CI: 18.67-39.73) months for the observation group, and 40.03 (95% CI: 36.31-50.62) for the adjuvant therapy group (*P* = .005; Supplementary Figure 3). Furthermore, the results of univariable and multivariable Cox regression analysis of RFS showed that the tumor size (HR: 2.475, 95% CI:1.541-3.975, *P* < .001), lymph node metastasis (HR: 1.765, 95% CI:1.132-3.203, *P* = .022), microvascular invasion (HR: 1.667, 95% CI:1.208-2.301, *P* = .002) and differentiation (HR: 2.329, 95% CI:1.526-3.554, *P* < .001) were independent prognostic factors in BTC. The results of the univariate and multivariate Cox regression analysis of OS showed that CEA (HR: 2.683, 95% CI:1.483-4.854, *P* = .001), tumor size (HR: 2.011, 95% CI:1.094-3.697, *P* = .025), lymph node metastasis (HR: 2.216, 95% CI:1.040-4.718, *P* = .039), differentiation (HR: 2.077, 95% CI:1.193-3.614, *P* = .010), and adjuvant therapy (HR: 2.560, 95% CI:0.395-0.793, *P* = .001) were independent prognostic factors in BTC (see Table 2 for details).

**Table 2.** Univariable and multivariable cox regression analysis of RFS and OS of 219 BTC patients.

Variables	RFS				OS			
	Univariate Hazard ratio (95% CI)	*P*-value	Multivariate Hazard ratio (95% CI)	*P*-value	Univariate Hazard ratio (95% CI)	*P*-value	Multivariate Hazard ratio (95% CI)	*P*-value
*Age, years*								
≤60 vs >60	1.122 (0.761-1.655)	.651	-	-	1.082 (0.656-1.785)	.756	-	-
*Sex*								
Male vs female	1.002 (0.686-1.462)	.993	-	-	1.055 (0.637-1.746)	.836	-	-
*Tumor location*								
iCCA vs dCCA vs pCCA vs GBC	0.923 (0.790-1.080)	.318	-	-	1.006 (0.823-1.229)	.955	-	-
*CA19-9, U/mL*								
≤37 vs >37	1.599 (1.092-2.342)	***.016***	1.109 (0.722-1.703)	.636	2.205 (1.305-3.725)	***.003***	1.136 (0.616-2.096)	.684
*CEA, ng/mL*								
≤ 5 > 5	1.824 (1.217-2.734)	***.004***	1.554 (0.975-2.476)	.064	3.223 (1.952-5.320)	***<.001***	2.683 (1.483-4.854)	***.001***
*Tumor size, cm*								
≤3 vs >3	2.546 (1.624-3.992)	***<.001***	2.475 (1.541-3.975)	***<.001***	2.458 (1.374-4.397)	***.002***	2.011 (1.094-3.697)	***.025***
*Tumor number*								
Single vs Multiple	1.337 (0.674-2.650)	.406	-	-	0.591 (0.185-1.888)	.375	-	-
*TNM stage*								
I + II vs III	2.354 (1.608-3.445)	***<.001***	1.663 (0.828-3.342)	.153	2.649 (1.608-4.365)	***<.001***	1.303 (0.534-3.179)	.560
*T stage*								
1 + 2 vs 3 + 4	1.827 (1.218-2.739)	***.004***	1.105 (0.648-1.884)	.715	2.050 (1.230-3.416)	***.006***	1.228 (0.599-2.517)	.574
*Lymph node metastasis*								
No vs Yes	2.471(1.670-3.657)	***<.001***	1.765 (1.132-3.203)	***.022***	2.516 (1.514-4.418)	***<.001***	2.216 (1.040-4.718)	***.039***
*Microvascular invasion*								
No vs Yes	1.457 (1.064-1.995)	***.019***	1.667 (1.208-2.301)	***.002***	1.456 (0.965-2.196)	.074	-	-
*Perineural invasion*								
No vs Yes	0.854 (0.583-1.249)	.854	-	-	0.778 (0.476-1.272)	.317	-	-
*Differentiation*								
Well/Moderate vs Poor/Undifferentiated	2.285 (1.543-3.384)	***<.001***	2.329 (1.526-3.554)	***<.001***	2.506 (1.514-4.148)	***<.001***	2.077 (1/193-3.614)	***.010***
*Margin status*								
R0 vs R1	1.644 (1.043-2.593)	***.032***	1.039 (0.622-1.737)	.883	2.059 (1.179-3.593)	***.011***	1.147 (0.606-2.170)	.673
*Adjuvant therapy*								
Observation vs chemotherapy vs chemoimmunotherapy	0.858 (0.680-1.082)	.195	-	-	0.609 (0.440-0.841)	***.003***	0.560 (0.395-0.793)	***.001***

**Figure 3.**
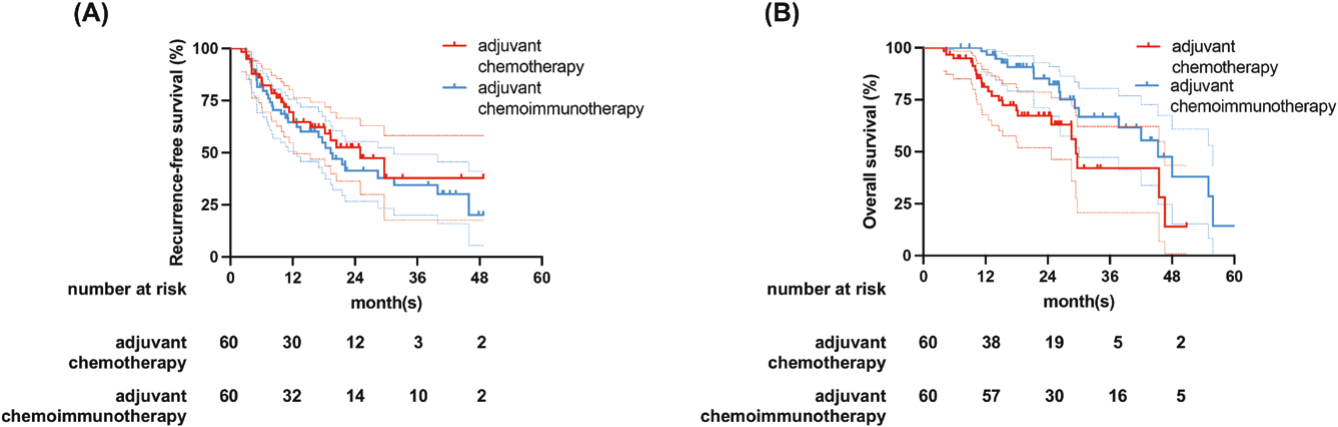
Kaplan–Meier curves for RFS (A) and OS (B) in the PSM cohort of adjuvant chemotherapy and adjuvant chemoimmunotherapy. RFS, recurrence-free survival; OS, overall survival.

To investigate the efficacy of adjuvant chemotherapy and adjuvant chemoimmunotherapy in the postoperative adjuvant treatment of BTC, we performed a PSM analysis in the 2 groups of patients in a 1:1 ratio (see Table 3 for details). After PSM, each group consisted of 60 patients. The median RFS was 22.03 (95% CI: 15.06-36.67) months for the adjuvant chemotherapy group, 19.87 (95% CI: 14.42-24.43) for the adjuvant chemoimmunotherapy group, respectively (*P* = .350). The median OS was 29.40 (95% CI: 21.73-31.07) months for the adjuvant chemotherapy group and 45.27 (95% CI: 37.58-51.78) for the adjuvant chemoimmunotherapy group, respectively (*P* = .015; Figure 3A, B). Also, the results of univariable and multivariable Cox regression analysis of RFS showed that the tumor size (HR: 3.803, 95% CI: 1.884-7.677, *P* < .001), lymph node metastasis (HR: 3.936, 95% CI: 1.604-7.828, *P* = .003) and differentiation (HR: 2.732, 95% CI: 1.515-4.893, *P* < .001) were independent prognostic factors in adjuvant therapy group. The results of univariable and multivariable cox regression analysis of OS showed that lymph node metastasis (HR: 2.700, 95% CI: 1.372-5.321, *P* < .001), differentiation (HR: 4.043, 95% CI: 1.808-9.040, *P* < .001) and adjuvant therapy (HR: 0.450, 95% CI: 0.222-0.909, *P* = .026) were independent prognostic factors in adjuvant therapy group (see Table 4 for details).

**Table 3.** The baseline patient characteristics of adjuvant chemotherapy and adjuvant chemoimmunotherapy patients.

Variables	Before PSM			After PSM		
	Adjuvant chemotherapy (*N* = 69)	Adjuvant chemoimmunotherapy (*N* = 63)	*P*-value	Adjuvant chemotherapy (*N* = 60)	Adjuvant chemoimmunotherapy (*N* = 60)	*P*-value
*Age, years*						1.000
≤60	29	26	.930	26	26	
>60	40	37		34	34	
*Sex*						.716
Male	33	27	.568	29	27	
Female	36	36		31	33	
*Tumor location*						.208
iCCA	32	36	.053	32	35	
pCCA	16	11		12	10	
dCCA	6	5		6	5	
GBC	15	11		10	10	
*CA19-9, U/mL*						.715
≤37	34	29	.710	31	28	
>37	35	34		29	32	
*CEA, ng/mL*						1.000
≤5	52	50	.585	48	47	
>5	17	13		12	13	
*Tumor size, cm*						.560
≤3	19	24	.198	18	21	
>3	50	39		42	39	
*Tumor number*						.188
Single	61	60	.158	53	57	
Multiple	8	3		7	3	
*TNM stage*						.714
I + II	41	35	.655	35	33	
III	28	28		25	27	
*T stage*						.688
1 + 2	48	46	.663	41	44	
3 + 4	21	17		19	16	
*Lymph node metastasis*						.235
No	53	39	.064	45	38	
Yes	16	24		15	22	
*Microvascular invasion*						1.000
No	54	49	.947	47	48	
Yes	15	14		13	12	
*Perineural invasion*						1.000
No	37	30	.601	30	31	
Yes	32	33		30	29	
*Differentiation*						.581
Well/moderate	28	29	.599	24	28	
Poor/undifferentiated	41	34		36	32	
*Margin status*						.616
R0	60	50	.254	52	49	
R1	9	13		8	11	

**Table 4.** Univariable and multivariable COX regression analysis of RFS and OS of 120 adjuvant treatment patients.

Variables	RFS				OS			
	Univariate Hazard ratio (95% CI)	*P*-value	Multivariate Hazard ratio (95% CI)	*P*-value	Univariate Hazard ratio (95% CI)	*P*-value	Multivariate Hazard ratio (95% CI)	*P*-value
*Age, years*								
≤60 vs >60	1.666 (0.984-2.822)	.057	-	-	1.500 (0.785-2.866)	.219	-	-
*Sex*								
Male vs Female	0.974 (0.583-1.628)	.921	-	-	1.323 (0.665-2.634)	.426	-	-
Tumor location								
iCCA vs dCCA vs pCCA vs GBC	0.924 (0.729-1.172)	.517	-	-	1.269 (0.964-1.669)	.089	-	-
*CA19-9, U/mL*								
≤37 vs >37	1.398 (0.834-2.344)	.204	-	-	1.693 (0.874-3.279)	.119	-	-
*CEA, ng/mL*								
≤ 5 > 5	1.909 (1.081-3.371)	***.026***	1.972 (1.103-3.524)	.022	1.918 (0.962-3.825)	.064	-	-
*Tumor size, cm*								
≤3 vs >3	3.333 (1.695-6.554)	***<.001***	3.803 (1.884-7.677)	***<.001***	1.953 (0.933-4.089)	.076	-	-
*Tumor number*								
Single vs multiple	1.741 (0.742-4.083)	.202	-	-	0.662 (0.158-2.768)	.572	-	-
*TNM stage*								
I + II vs III	2.758 (1.619-4.697)	***<.001***	1.052 (0.367-3.014)	.925	2.281 (1.188-4.381)	***.013***	2.291 (0.983-5.339)	.055
*T stage*								
1 + 2 vs 3 + 4	2.277 (1.340-3.867)	***.002***	1.332 (0.655-2.709)	.428	2.618 (1.351-5.076)	***.004***	2.056 (0.838-5.046)	.116
*Lymph node metastasis*								
No vs Yes	3.684 (2.157-6.291)	***<.001***	3.936 (1.604-7.828)	***.003***	2.375 (1.237-4.560)	***.009***	2.700 (1.372-5.321)	***<.001***
*Microvascular invasion*								
No vs Yes	0.834 (0.478-1.455)	.522	-	-	0.696 (0.362-1.339)	.278	-	-
*Perineural invasion*								
No vs Yes	0.926 (0.547-1.567)	.775	-	-	0.775 (0.236-2.543)	.674	-	-
*Differentiation*								
Well/moderate vs poor/undifferentiated	2.381 (1.383-4.099)	***.002***	2.723 (1.515-4.893)	***<.001***	4.222 (1.937-9.199)	***<.001***	4.043 (1.808-9.040)	***<.001***
*Margin status*								
R0 vs R1	1.377 (1.177-2.802)	***.011***	0.531 (0.241-1.172)	.117	1.554 (0.638-3.789)	.332	-	-
*Adjuvant therapy*								
Chemotherapy vs chemoimmunotherapy	1.284 (0.760-2.168)	.350	-	-	0.423 (0.212-0.846)	***.015***	0.450 (0.222-0.909)	***.026***

### Adverse events

The details of drug-related adverse events (AE) are shown in Supplementary Table 1. The most common AE were any grade of anemia, neutropenia, thrombocytopenia, and leukopenia. Most AEs were mild to moderate (grade 1 or 2) and resolved with appropriate treatment. It is worth noting that the most significant difference between the 2 groups is the incidence of rash. The incidence of rash in the adjuvant chemoimmunotherapy group was significantly higher than in the adjuvant chemotherapy group (*P* < .001), but the incidence of rash in grade 3/4 was low, and symptomatic treatment was able to alleviate it (see Supplementary Table 1 for more details).

## Discussion

This study retrospectively analyzed the data of 219 postoperative patients with BTC, clarified the need for postoperative adjuvant therapy, and compared the effects of different adjuvant therapy methods on the prognosis. Adjuvant chemoimmunotherapy was found to significantly prolong the OS of patients. Chemoimmunotherapy is routinely recommended for postoperative patients with BTC.

Although there have been some research reports that question the value of postoperative adjuvant therapy, there are more studies that support routine adjuvant therapy after BTC surgery to reduce tumor recurrence and prolong patient survival.^15^ Especially for patients with some risk factors for recurrence, such as lymph node positivity and late staging, postoperative adjuvant therapy is more necessary. Currently, the selection of postoperative chemotherapy regimens is still relatively limited, mainly based on the gemcitabine chemotherapy regimen, the 5-Fu chemotherapy regimen, and the S-1 treatment.^16-18^ Due to its oral nature, convenience, relatively mild adverse reactions, and higher execution rate, S-1 is widely used among the population. In this study, S-1 consumption was up to 47.73% between chemotherapy regimens. However, due to the small sample size, we did not compare the differences between S-1 and other chemotherapy regimens. Also, due to the limited number of patients and the fact that the combination of chemotherapy and immunotherapy drugs was determined by multiple factors, it was difficult to compare and evaluate this detail. We also await some new clinical evidence to provide new options for postoperative chemotherapy regimens for BTC.

The emergence of immunotherapy is a milestone in tumor treatment, with significant therapeutic effects in various solid tumors. In the experience of BTC application, the efficacy of immunotherapy is relatively less remarkable.^19,20^ However, more and more studies are trying to improve the prognosis of BTC by combining immunotherapy with other treatment methods, and expanding the application of immunotherapy in BTC, including various treatment modes such as neoadjuvant therapy, postoperative adjuvant therapy, first-line treatment for advanced BTC.^21-24^ In this study, the results showed that combination immunotherapy with adjuvant chemotherapy could significantly prolong the median OS from 29.4 to 45.27 months. Although the impact on RFS in patients was not significant, it could bring benefits to OS without significantly increasing serious adverse reactions. This also supported chemoimmunotherapy as an optional strategy for postoperative adjuvant therapy in patients with BTC.

Overall, the prognosis for BTC patients is poor, which is related to the characteristics of BTC tumor cells. In this study, the results showed that tumor size, lymph node metastasis, microvascular invasion, and differentiation were independent prognostic factors for RFS in BTC. CEA, tumor size, lymph node metastasis, differentiation, and adjuvant therapy were independent prognostic factors for OS in BTC. For patients who received adjuvant therapy, tumor size, lymph node metastasis, and differentiation were independent prognostic factors and independent prognostic factors for RFS in adjuvant BTC patients. Lymph node metastasis, differentiation, and adjuvant therapy were independent prognostic factors for OS in adjuvant patients with BTC. There were some results with a little difference before and after PSM. It is believed that the results after PSM are more reliable and more patients in this situation will be collected to validate these findings in future work.

On the basis of these results, it is further confirmed that lymph node metastasis and poor differentiation are important factors that affect the prognosis of BTC. Exploring the molecular mechanisms underlying why BTC is prone to lymph node metastases may be the key to improving the prognosis of BTC.^25^ Furthermore, lymph node metastasis has been clinically recognized to have a significant impact on the prognosis of BTC. Preoperative imaging evaluation and prediction provide some clues for an accurate diagnosis of BTC.^26,27^ The induction and summary of molecular characteristics of patients with lymph node metastases provide some basis for the development of targeted therapy.^28,29^

In terms of adverse reactions, both adjuvant groups had a higher incidence of hematologic toxicity and hand-foot syndrome, which were common adverse reactions of chemotherapy drugs. The incidence rate for grade 3/4 was very low, indicating good safety and tolerability. For the chemoimmunotherapy group, the rash was more common and could be relieved and maintained for more than 6 cycles after symptomatic treatment. In general, the safety of chemoimmunotherapy was acceptable and could significantly prolong the overall survival of BTC. These data provide a basis for an additional prospective clinical trial to test the efficacy of chemoimmunotherapy in the adjuvant setting.

## Supplementary Material

oyaf163_suppl_Supplementary_Figures_1-3

oyaf163_suppl_Supplementary_Tables_1

## Data Availability

Data and materials were included in the manuscript.
