# Supplementary material for: Postoperative adjuvant chemotherapy and chemoimmunotherapy after radical resection for biliary tract cancer: a retrospective study
Source: Oncologist. 2025 Jun 21;30(6):oyaf163. doi: 10.1093/oncolo/oyaf163 (PMC12203059; doi:10.1093/oncolo/oyaf163)
Supplement: oyaf163_suppl_Supplementary_Figures_1-3 [file oyaf163_suppl_supplementary_figures_1-3.docx]

Supplemental Figure 1 Kaplan–Meier curves for RFS (A) and OS (B) in the primary cohort of 219 patients. RFS: recurrence-free survival, OS: overall survival.

Supplemental Figure 2 Kaplan–Meier curves for RFS (A) and OS (B) before PSM cohort of surgery alone and adjuvant chemotherapy. Kaplan–Meier curves for RFS (C) and OS (D) after PSM cohort of surgery alone and adjuvant chemotherapy. RFS: recurrence-free survival, OS: overall survival.

Supplemental Figure 3 Kaplan–Meier curves for RFS (C) and OS (D) in the primary cohort of surgery alone, adjuvant therapy. RFS: recurrence-free survival, OS: overall survival.
