# Supplementary material for: Postoperative adjuvant chemotherapy and chemoimmunotherapy after radical resection for biliary tract cancer: a retrospective study
Source: Oncologist. 2025 Jun 21;30(6):oyaf163. doi: 10.1093/oncolo/oyaf163 (PMC12203059; doi:10.1093/oncolo/oyaf163)
Supplement: oyaf163_suppl_Supplementary_Tables_1 [file oyaf163_suppl_supplementary_tables_1.docx]

**Supplemental Table 1 Postoperative adjuvant therapy alone or in combination with immunotherapy-related adverse reactions after PSM.**

| **Adverse events** | **Any grade** | | |  | **Grade 3/4** | | |
| --- | --- | --- | --- | --- | --- | --- | --- |
|  | **Adjuvant chemotherapy** | **Adjuvant chemoimmunotherapy** | **P value** |  | **Adjuvant chemotherapy** | **Adjuvant chemoimmunotherapy** | **P value** |
| Leukopenia | 15 (25.00%) | 19 (31.67%) | 0.544 |  | 2 (3.33%) | 4 (6.67%) | 0.679 |
| Neutropenia | 14 (23.33%) | 26 (43.33%) | 0.163 |  | 7 (11.67%) | 9 (15.00%) | 0.789 |
| Anemia | 27 (45.00%) | 28 (46.67%) | >0.999 |  | 6 (10.00%) | 7 (11.67%) | >0.999 |
| Thrombocytopenia | 22 (36.67%) | 25 (41.67%) | 0.709 |  | 4 (6.67%) | 5 (8.33%) | >0.999 |
| AST/ALT elevation | 8 (13.33%) | 10 (16.67%) | 0.799 |  | 0 | 0 | - |
| Proteinuria | 4 (6.67%) | 6 (10.00%) | 0.743 |  | 1 (1.67%) | 2 (3.33%) | >0.999 |
| Hematuresis | 3 (5.00%) | 4 (6.67%) | >0.999 |  | 0 | 0 | - |
| Creatinine/BUN elevation | 6 (10.00%) | 7 (11.67%) | >0.999 |  | 1 (1.67%) | 2 (3.33%) | >0.999 |
| Rash | 1 (1.67%) | 18 (30%) | **<0.001** |  | 0 | 4 (6.67%) | 0.119 |
| Fatigue | 4 (6.67%) | 5 (8.33%) | >0.999 |  | 0 | 0 | - |
| Hand-foot syndrome | 22 (36.67%) | 24 (40.00%) | 0.851 |  | 2 (3.33%) | 4 (6.67%) | 0.679 |
